# Supplementary material for: Enabling high throughput deep reinforcement learning with first principles to investigate catalytic reaction mechanisms
Source: Nat Commun. 2024 Jul 25;15:6281. doi: 10.1038/s41467-024-50531-6 (PMC11282263; doi:10.1038/s41467-024-50531-6)
Supplement: Supplementary file 1 — Supplementary Information [file 41467_2024_50531_MOESM1_ESM.pdf]

Supplementary Information

Enabling High Throughput Deep Reinforcement Learning with First Principles to Investigate Catalytic Reaction Mechanisms

Tian Lan[1], Huan Wang[1] and Qi An[2*]

[1]Salesforce A.I. Research, Palo Alto, CA 94301, USA

[2]Department of Materials Science and Engineering, Iowa State University, Ames, IA 50011, USA

[*]Corresponding author E-mail: qan@iastate.edu

The Supplementary Information includes (1) definition of action and reward system in reinforcement learning (RL), (2) exploring NH_2 hydrogenation reaction using high density mesh, (3) the N diffusion pathway identified by RL and nudged elastic band (NEB), (4) free energy estimation at T=673K and P=20atm for the Langmuir-Hinshelwood (LH) and Eley-Rideal (ER) configurations, and (5) comparison of high-throughput deep reinforcement learning with first principles (HDRL-FP) framework with other methods. It also includes Table S1 (The cutoff distance to determine whether the migrated H or H atom moves to the forbidden region or not), Table S2 (The hyperparameters used in the RL model), and Figure S1-S6.

1. Definition of Environment in RL

1.1 Action definition

In conducting the density functional theory (DFT) calculations, we employed a periodic slab model consisting of six layers of Fe atoms, the same as we used in previous studies.[1,2] Each layer comprises a unit cell with dimensions of (2 × 2) and an area (8.01 × 6.94 $Å^2$), accommodating four Fe atoms per layer. To mitigate potential interactions between the replicated cells in DFT calculations due to periodicity, we incorporated a 10 Å vacuum space in the z direction, leading to a cell length of 14.81 Å along the z-axis. The top three layers underwent relaxation, while the bottom three layers remained fixed. To define actions in the RL process within this supercell, we constructed a three-dimensional grid with dimensions 20 × 18 × 50 inside the simulation cell. This led to a grid space of less than 0.40 Å in each direction. In our methodology, we adopted a grid spacing of 0.4 Å, significantly smaller than the equilibrated structure's smallest bond distance, N-H, which is 1.02 Å. This chosen grid spacing, at 0.4 Å, facilitates the creation of a smooth potential energy surface. It ensures small energy fluctuations between neighboring grid points, as demonstrated in Figures 3(a) and 3(d). However, it is important to note that increasing the density of grid points substantially increases computational demands, primarily due to the computational cost of DFT simulations. To demonstrate that the 0.4 Å grid space reaches convergence, we conducted the calculations on the NH_2 hydrogenation reaction (section 2.3 in the main text) and obtained the consistent reaction path, which is in detailed discussion in section 6 of SI. The migrating atom, either H or N, navigates through each grid in the RL calculations, thereby mapping out the potential energy landscape (PEL) using the electronic energy derived from the DFT simulations. The movements of migrating atoms in these grids define six actions: (1) forward, (2) backward, (3) up, (4) down, (5) left, and (6) right.

1.2 Reward Definition

In this study, we utilized a simple reward definition in the context of RL calculations, favoring a negative reward system to facilitate identifying the shortest reaction pathway with the least energy barrier. We employed a reference configuration, 2N_NH_2_2H, from our previous work,[1] as displayed in Figure S1 of the SI. We defined the reward as $r = -\Delta E/E_0$, where ΔE (measured in eV) is the difference in electronic energy between the reference state and the examined configuration within the RL, derived from density functional theory (DFT). As part of our exploration of the configuration space in RL, we performed self-consistent field (SCF) calculations for each configuration, explicitly prohibiting atomic relaxation. In this context, E_0 acts as a reward scaling factor, which we set to 20 eV in this study. For the initial reactant in RL, we shifted the surface NH_2 molecule from the top-shallow (TS) site to the top site, consistent with the final product. This adjustment resulted in a higher energy compared to the reference state.

It is important to note that during the chemical reaction, atoms tend to avoid excessive proximity due to strong repulsion arising from the kinetic energy of electrons. As such, if the distance between the migrating atom and its nearest neighbor is less than the cutoff distance listed in Table S1 of the SI, we assign a maximum negative reward (-1). This cutoff distance is consistently less than 70% of the equilibrium bond distance between the migrated H (or N) atom and other atoms. When the H (or N) atom is approximately 70% of the equilibrium bond distance from other atoms, the energies of these configurations range from 10 to 20 eV, according to DFT calculations. Consequently, we selected E_0 = 20 eV for consistency when the H (or N) atom migrates into the restricted region.

2. Exploring NH_2 Hydrogenation reaction using high density mesh

To demonstrate the convergence of using 0.4 Å grid spacing in our HDRL-FP approach to study the H-B chemical reactions on Fe(111) surface, we conduct the calculations with a 0.2 Å grid spacing on the reaction from 2N_NH_2_2H to 2N_NH_3_H, which is discussed in section 2.3 of main text. It is important to note that the doubling of the mesh points effectively increased the grid density by eightfold. Given that each atom has six different action choices at each grid vertex, this leads to an exponential increase in the number of possible paths, roughly by a factor of 6^M, where M could be close to 7, signifying a significant increase. While the complexity can be mitigated by selectively increasing density in certain regions, or by resuming finer exploration from the previous learnt policy tackling the 0.4 Å scenario, we opted to challenge our framework and test it under the highest level of complexity for this problem. Consequently, we uniformly increased the grid density from 0.4 Å spacing to 0.2 Å and employed RL to directly explore this new environment from scratch.

Similar to our study in the main text, we explore two potential reaction mechanisms: (1) the Langmuir-Hinshelwood (LH) mechanism, where both reactants are present on the catalytic surface, and (2) the Eley-Rideal (ER) mechanism, where one hydrogen atom originates from the gas phase. Our DFT calculations show that 0.4 Å grid spacing generates the smooth potential energy surface (PES) along the reaction pathway, as evidenced by the absence of significant energy fluctuations between adjacent grid points (Figure 3(d) of main text). Therefore, we generate the energetic data for 0.2 Å grid based on the interpolation of 0.4 Å DFT data. To perform uniform interpolation for the mesh grid, transitioning from 0.4 Å to 0.2 Å, we employed proximal interpolation based on the nearest neighbor method, commonly utilized in 3D rendering to determine color values for textured surfaces. In our scenario, three categories of empty sites necessitate interpolation: (1) Midpoints of grid edges (edge center of two neighboring points of

0.4 Å griding): We compute the average value of the two vertices comprising the edge; (2) Centers of grid surfaces (face center of 4 neighboring points of 0.4 Å griding): We calculate the average value of the four vertices composing each surface; and (3) Centers of grid cubic cells (body center of 8 neighboring grids of 0.4 Å griding): We determine the average value of the eight vertices constituting each cell.

To demonstrate that the interpolation provides an accurate description of PES, we did the DFT calculations along both ER and LH reaction paths. As shown in Figure S2, the interpolation along both reaction paths agrees very well with the complete DFT calculations. Therefore, our new RL calculations of 0.2 Å grid are based on this new PES data. Figure S3(a, b) illustrates the convergence speed when processing the LH and ER reactions, respectively, with n = 500 environment replicas running in parallel. Our findings reveal that in all separate runs, LH simulations reach the same global optimum within 135 minutes (vs. 45 minutes under the 0.4 Å setting), whereas ER simulations achieve the global optimum within 90 minutes (vs. 40 minutes under the 0.4 Å setting). These experiments once again underscore the robustness and consistent convergence of our method. The convergence of RL is slower compared to previous calculations of 0.4 Å grid spacing, owing to the exponential increase of the complexity. However, the obtained reaction paths for both ER and LH mechanisms are consistent with the prediction of 0.4 Å grid spacing. These new calculations demonstrate that our previous calculation with 0.4 Å grid spacing obtains useful information with much reduced computational cost.

3. N diffusion pathway from HDRL-FP and NEB

To demonstrate the generalizability of the high-throughput deep reinforcement learning with first principles (HDRL-FP) framework, we applied it to evaluate nitrogen (N) diffusion on a bare Fe(111) surface. This test employs a reward system similar to that used for H migration, with a

scaling factor E_0 = 20 eV. We introduced a cutoff distance to prevent N from closely contacting neighboring atoms during movement, details of which are presented in Table S1 of the SI. The reference state was based on an optimized 1N configuration on the Fe(111) surface, characterized by an electronic energy of -193.73 eV. The diffusion pathway and the potential energy of each intermediate state along the path are visualized in Figure S4(a,b) of the SI. The N atom migrates from the original bridge site to the end of the bridge site, navigating between the top-layer Fe atom and those in the sublayer and 3rd layer. Notably, the highest energy barrier emerges as the N atom transitions through the intermediate region between the top layer and sublayer, along the PEL. This barrier is approximately 8.60 eV in this region, a value that is higher than expected due to the constraints placed on Fe atom relaxation in the RL calculations. It should be noted that the final N bridge state is energetically higher than the original state (Figure S4b). This discrepancy arises because the RL was conducted based on the relaxed structure for the original N position, but the Fe atoms surrounding the N in the final state were not relaxed, resulting in a higher energy state.

We conducted Nudged Elastic Band (NEB) calculations for the same N diffusion process. As shown in Figure S5 of SI, the diffusion path aligns with the RL prediction, presenting two energy barriers during the diffusion process. The maximum energy barrier derived from NEB calculation is 0.85 eV for N diffusion, which is significantly lower compared to the RL. In the RL calculations, we kept all atoms fixed except for the migrating N atom. Consequently, the unrelaxed PEL generally presents higher energy compared to the relaxed structure in the NEB calculation.

4. Free energy estimation at 673 K

In the H migration from the reaction $2N\_NH_2\_2H$ to product $2N\_NH_3\_H$, the Eley-Rideal (ER) and Langmuir-Hinshelwood (LH) mechanisms share the same transition state. To ascertain which mechanism is more probable under realistic conditions, we estimate the free energy of reactants for both mechanisms. We computed the free energy correction at $T = 673$ K and $P = 20$ atm ($P_{H2} = 15$ atm and $P_{N2} = 5$ atm) for consistency with previous theoretical studies[1,2] and Fe single crystal experiment[3.4]. For LH mechanism, we selected the newly identified configuration (Figure S2) as the reactant and estimate the free energy correction to be -1.09 eV. The electronic energy for this configuration is -228.15 eV, making the total free energy for the configuration with two H atoms adsorbed on the surface -229.24 eV. For the ER mechanism, we consider a $2N\_NH_2\_H$ slab with half an H_2 molecule in the gas phase. The electronic energy and free energy correction for the $2N\_NH_2\_H$ slab is -224.24 eV and -1.29 eV, respectively, leading to a total free energy of -225.54 eV. The free energy for the H_2 molecule at $T = 673$ K and $P = 20$ atm is -6.77 eV.[5] Based on DFT calculations (PBE-D3), the binding energy of H_2 molecule is 4.57 eV. Therefore, the free energy for the ER configuration is estimated to be $-225.54 - 0.5 \times 6.77 + 0.5 \times 4.57 = -226.64$ eV. The ER reactant presents a significantly higher free energy compared to the LH reactant, suggesting that the LH mechanism is more plausible under the experimental condition ($T = 673$ K and $P = 20$ atm).

5. Comparison of HDRL-FP framework with other methods

Our HDRL-FP approach fundamentally differs from other methods used to identify the reaction path, such as the artificial force induced reaction (AFIR) method and NEB method. The AFIR approach involves applying a constant force to bring reactant molecules together in order to identify the reaction path and transition state.[5,6] This approach focuses on determining the reaction path solely by providing multiple reactant species, without prior knowledge of the

reaction mechanisms. Similar to NEB approach, the AFIR approach is effective in determining the reaction path in a relatively simple reaction step. In contrast, the HDRL-FP approach constructs the reaction path directly from PEL obtained through DFT and efficiently explores the full PEL. With this approach, the reaction path can be predicted with only the knowledge of the initial and final states of a chemical reaction, even without detailed understanding of the reaction mechanisms.

Table S1. Cutoff distances used to determine whether the migrated H or N atom has moved into the forbidden region (proximity to the nearest neighbor atoms)

Bond type	Cutoff distance (Å)
H-H	0.5
N-H	0.7
Fe-H	1.0
N-N	0.8
Fe-N	1.0

Table S2. The hyperparameters used in the deep RL model.

Hyperparameters	Values
Training Algorithm	Proximal Policy Optimization (PPO)
Episode Length per Environment	500
Number of Environment Replicas	100 or 500
Training Batch Size	(100 or 500) X 500
Learning Rate	0.001
Entropy Regulation Coefficient	0.1
Reward Discount Factor	0.99
Number of Fully-Connected Layers	2
Fully-Connected Layer Dimension	50
Fully-Connected Layer Activation	ReLU
Output Layer Dimension (Policy)	6
Output Layer Activation (Policy)	Softmax
Output Layer Dimension (Value)	1
Policy/Value Networks Shared Weights	True

Figure S1

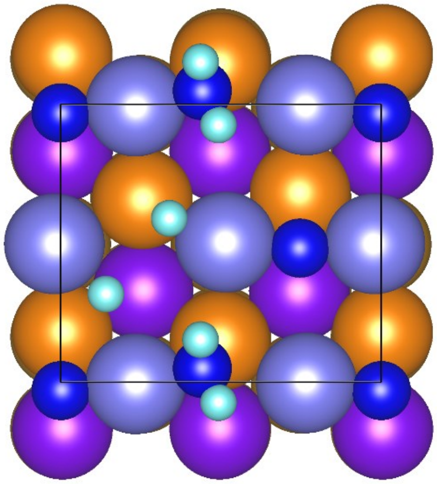

Figure S1. The reference configuration 2N-NH_2-2H that is used for reward calculations in the deep RL.[1] In this configuration, one H is located in shallow-deep (SD) site and the other H is in the top-shallow (TS) site. This configuration is from Ref. 1 with the electronic energy of -227.993 eV from density functional theory (DFT) calculations. The Fe atoms in the top, sublayer, deep-layer, and 4th-6th layers are represented by light blue, orange, purple, and bronze spheres, respectively. N and H atoms are represented by blue and cyan spheres, respectively. The structure file is provided in Supplementary Data 1.

Figure S2

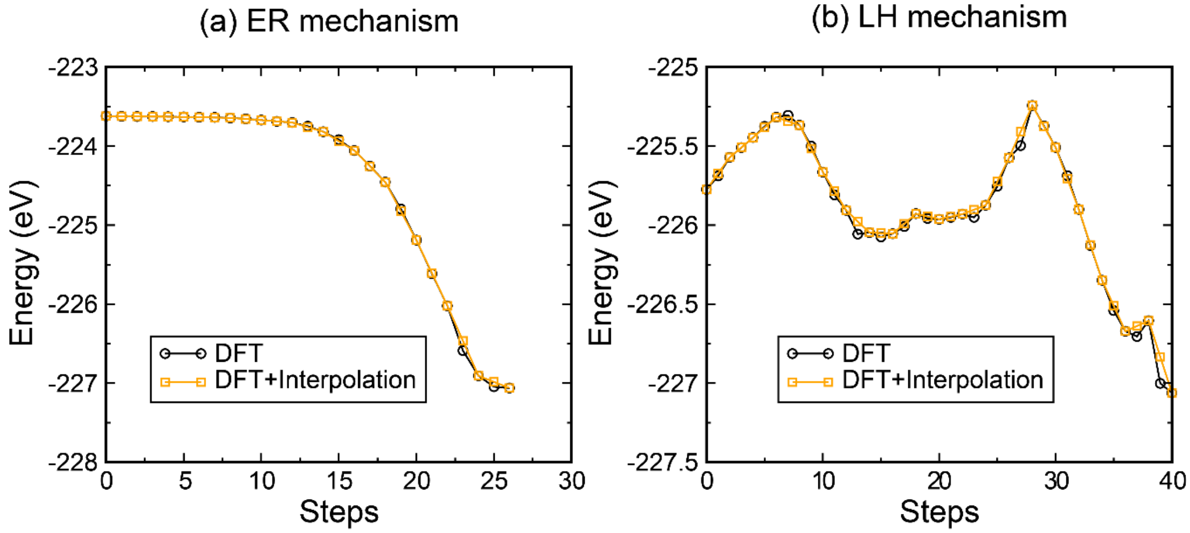

Figure S2. Comparison of direct DFT simulations with a 0.2 Å grid spacing versus interpolated data from 0.4 Å grid spacing DFT for: (a) the reaction pathway of H migration to NH_2, resulting in NH_3 formation, according to the Eley-Rideal mechanism; and (b) the reaction pathway for H migration to NH_2, resulting in NH_3 formation, via the Langmuir-Hinshelwood mechanism. The orange curves represent a combination of DFT data (for even steps) and interpolated data (for odd steps), as detailed in Section 3 of the SI. Source data are provided as a Source Data file.

Figure S3

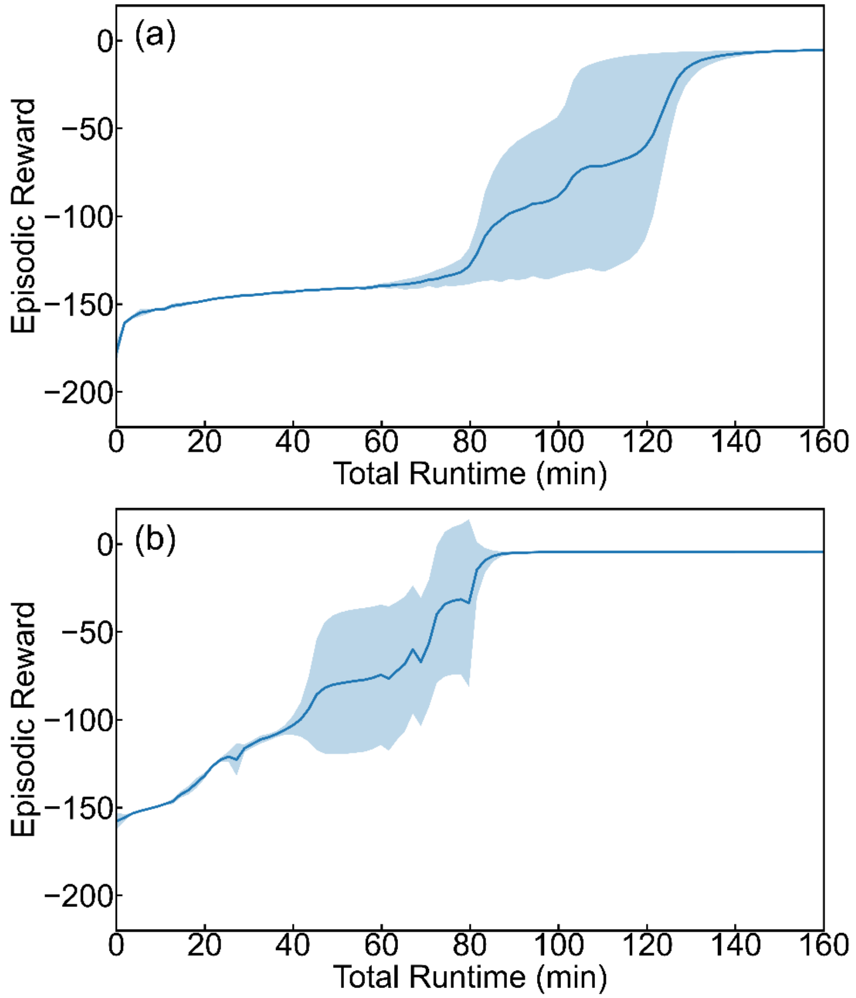

Figure S3: Convergence and learning speed, measured in total runtime using wall-clock minutes, for our framework with n=500 environment replicas under 0.2 Å setting applied to (a) Langmuir-Hinshelwood and (b) Eley-Rideal hydrogenation reaction of NH_2 to NH_3. The episodic reward is the mean accumulated reward that H atom actors collect from the initial to the terminal state of (a) Langmuir-Hinshelwood and (b) Eley-Rideal. For robustness, the depicted results are averaging over five independent runs from scratch with different initialization seeds and the same hyperparameters. The shadow regions represent the error bar (standard deviation) of five independent runs. Source data are provided as a Source Data file.

Figure S4

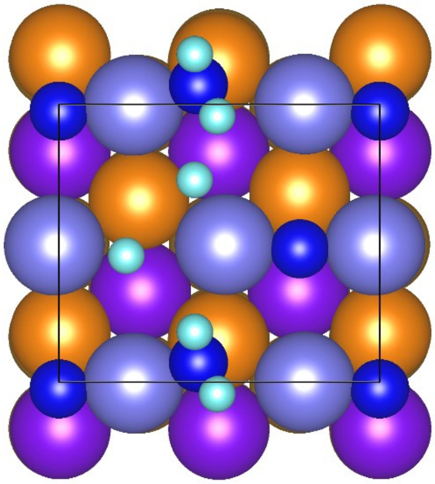

Figure S4. The newly identified 2N-NH_2-2H configuration, which has a lower energy compared to the configuration depicted in Figure S1.[4,5] This configuration has an electronic energy of -228.149 eV as determined by DFT, which is 0.156 eV lower than the configuration in Figure S1. In this configuration, both H atoms are situated in the top-shallow-deep (TSD) site. The Fe atoms in the top, sublayer, deep-layer, and 4th-6th layers are represented by light blue, orange, purple, and bronze spheres, respectively. N and H atoms are represented by blue and cyan spheres, respectively. The structure file is provided in Supplementary Data 1.

Figure S5

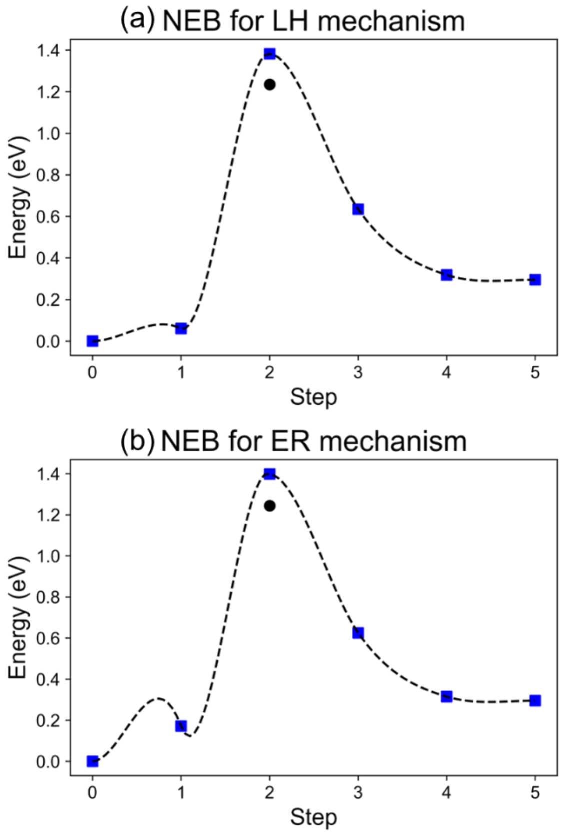

Figure S5. Results of the Nudged Elastic Band (NEB) calculation for the H migration from 2N_NH_2_2H to 2N_NH_3_H for the (a) Langmuir-Hinshelwood (LH) and (b) Eley-Rideal (ER) mechanisms. The blue squares represent data points from the Climbing Image Nudged Elastic Band (CI-NEB) method, and the black circles represent data points obtained from the dimer method following NEB calculations. Source data are provided as a Source Data file.

Figure S6

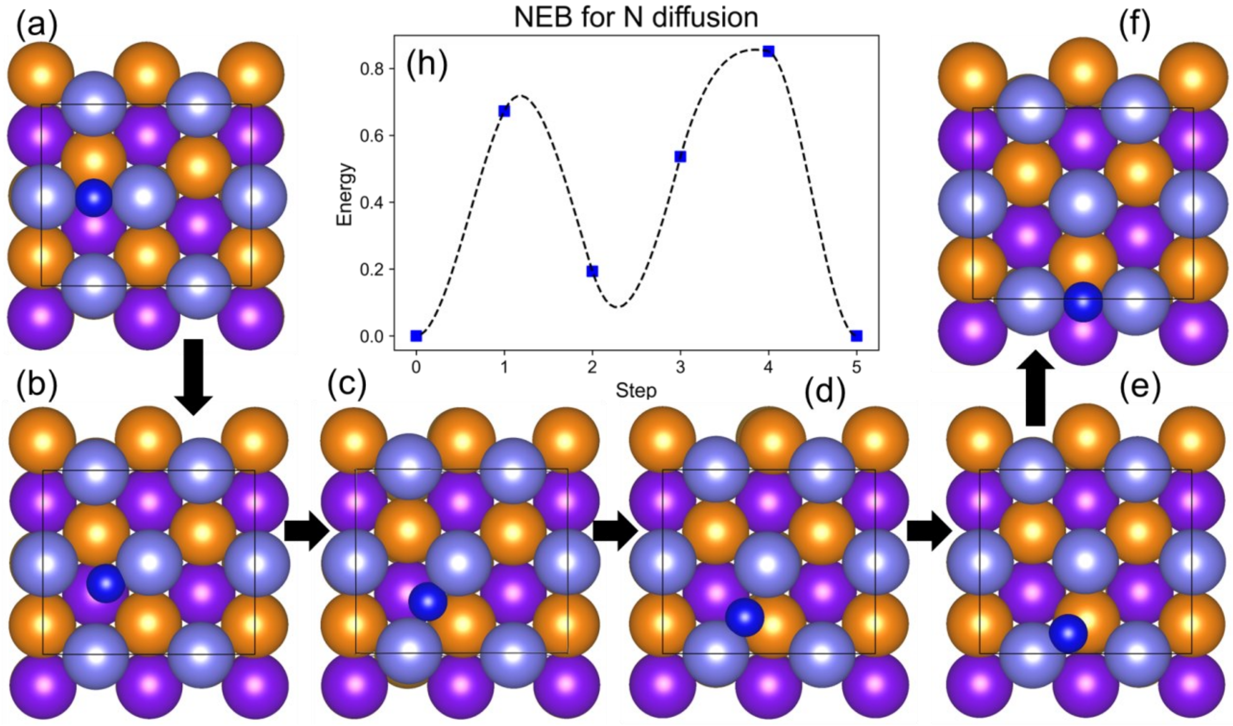

Figure S6. The NEB calculation for the N diffusion between two bridge sites: (a)-(f) depict the configurations along the NEB path, while (h) represents the potential energy derived from NEB calculation. The blue squares represent data points from the Climbing Image Nudged Elastic Band (CI-NEB) method. Steps 0 and 5 correspond to the initial reactant and final product of the NEB calculations, respectively. Steps 1—4 correspond to configurations (b-e). The Fe atoms in the top, sublayer, deep-layer, and 4th-6th layers are represented by light blue, orange, purple, and bronze spheres, respectively. The N atom is represented by the blue sphere. Source data for (h) are provided as a Source Data file. The structure files are provided in Supplementary Data 1.

References

(1) Lan, T. & An, Q. Discovering catalytic reaction networks using deep reinforcement learning from first-principles *J. Am. Chem. Soc.* **143**, 16804–16812 (2021).

(2) Qian, J., An, Q., Fortunelli, A., Nielsen, R. J. & Goddard, W. A. III Reaction mechanism and kinetics for ammonia synthesis on the Fe(111) surface. *J. Am. Chem. Soc.* **140**, 6288–6297 (2018).

(3) Somorjai, G. A. & Materer, N. Surface structures in ammonia synthesis. *Top. Catal.* **1**, 215−231 (1994).

(4) Strongin, D. R., Carrazza, J., Bare, S. R. & Somorjai, G. A. The importance of C7 sites and surface roughness in the ammonia-synthesis reaction over iron. *J. Catal.* **103**, 213−215 (1987).

(5) Maeda, S. & Morokuma, K. A systematic method for locating transition structures of A+B\X type reactions. *J. Chem. Phys.* **132**, 241102 (2010).

(6) Zimmerman, P. M. Automated discovery of chemically reasonable elementary reaction steps. *J. Comput. Chem.* **34**, 1385–1392 (2013).
