# Supplementary material for: Enabling high throughput deep reinforcement learning with first principles to investigate catalytic reaction mechanisms
Source: Nat Commun. 2024 Jul 25;15:6281. doi: 10.1038/s41467-024-50531-6 (PMC11282263; doi:10.1038/s41467-024-50531-6)
Supplement: Supplementary file 3 — Description of Additional Supplementary File [file 41467_2024_50531_MOESM3_ESM.docx]

**Description of Supplementary Data file**

The Supplementary Data file includes the atomic structures for the density functional theory (DFT) calculations. Particularly, it includes

- H trajectory as H migrates from 2N_NH_2__2H to 2N_NH_3__H via Langmuir-Hinshelwood mechanism.
- H trajectory as H migrates from 2N_NH_2__2H to 2N_NH_3__H via Eley-Rideal mechanism.
- Nudged elastic band (NEB) reaction path (reactant, transition state and product) of hydrogen migration from 2N_NH_2__2H to 2N_NH_3__H via Langmuir-Hinshelwood mechanism.
- Nudged elastic band (NEB) reaction path (reactant, transition state and product) of hydrogen migration from 2N_NH_2__2H to 2N_NH_3__H via Eley-Rideal mechanism.
- The identified N diffusion path on the bare Fe(111) surface as determined by reinforcement learning (No N relaxation)
- The identified N diffusion path on the bare Fe(111) surface as determined by reinforcement learning (N relaxation)
- The N2 diffusion path (reactant, transition state, product) on the bare Fe(111) surface from one top site to another top site.
- The reference configuration 2N-NH_2_-2H for reward calculations in the reinforcement learning.
- The newly identified 2N-NH_2_-2H configuration, which has a lower energy compared to the reference configuration above.
- The Nudged elastic band path for the N diffusion between two bridge sites.
