# Supplementary material for: Enabling high throughput deep reinforcement learning with first principles to investigate catalytic reaction mechanisms
Source: Nat Commun. 2024 Jul 25;15:6281. doi: 10.1038/s41467-024-50531-6 (PMC11282263; doi:10.1038/s41467-024-50531-6)
Supplement: Supplementary file 5 — Reporting Summary [file 41467_2024_50531_MOESM5_ESM.pdf]

nature portfolio

Corresponding author(s): Qi An

Last updated by author(s): May 31, 2024

Reporting Summary

Nature Portfolio wishes to improve the reproducibility of the work that we publish. This form provides structure for consistency and transparency in reporting. For further information on Nature Portfolio policies, see our Editorial Policies and the Editorial Policy Checklist.

Statistics

For all statistical analyses, confirm that the following items are present in the figure legend, table legend, main text, or Methods section.

n/a	Confirmed	
☐	☒	The exact sample size (n) for each experimental group/condition, given as a discrete number and unit of measurement
☒	☐	A statement on whether measurements were taken from distinct samples or whether the same sample was measured repeatedly
☐	☒	The statistical test(s) used AND whether they are one- or two-sided<br>*Only common tests should be described solely by name; describe more complex techniques in the Methods section.*
☒	☐	A description of all covariates tested
☐	☒	A description of any assumptions or corrections, such as tests of normality and adjustment for multiple comparisons
☐	☒	A full description of the statistical parameters including central tendency (e.g. means) or other basic estimates (e.g. regression coefficient) AND variation (e.g. standard deviation) or associated estimates of uncertainty (e.g. confidence intervals)
☒	☐	For null hypothesis testing, the test statistic (e.g. F, t, r) with confidence intervals, effect sizes, degrees of freedom and P value noted<br>*Give P values as exact values whenever suitable.*
☒	☐	For Bayesian analysis, information on the choice of priors and Markov chain Monte Carlo settings
☐	☒	For hierarchical and complex designs, identification of the appropriate level for tests and full reporting of outcomes
☒	☐	Estimates of effect sizes (e.g. Cohen's d, Pearson's r), indicating how they were calculated

Our web collection on statistics for biologists contains articles on many of the points above.

Software and code

Policy information about availability of computer code

Data collection	We used Vienna Ab initio Simulation Package (VASP, with version 5.4) for density functional theory (DFT) calculations, VASP (v5.4) for nudged elastic band (NEB) and dimer calculations, and WarpDrive (v2.7) for reinforcement learning calculations. The multi-agent reinforcement learning code is explained in Ref. 22 and the WarpDrive (version 2.7) is available at https://github.com/salesforce/warp-drive . The environment code is available at https://github.com/salesforce/warp-drive/tree/master/example_envs .
Data analysis	In this study, we used VESTA (veresion 3.5.8) to analyze the data and results.

For manuscripts utilizing custom algorithms or software that are central to the research but not yet described in published literature, software must be made available to editors and reviewers. We strongly encourage code deposition in a community repository (e.g. GitHub). See the Nature Portfolio guidelines for submitting code & software for further information.

Data

Policy information about availability of data

All manuscripts must include a data availability statement. This statement should provide the following information, where applicable:

- Accession codes, unique identifiers, or web links for publicly available datasets
- A description of any restrictions on data availability
- For clinical datasets or third party data, please ensure that the statement adheres to our policy

All data generated in this study are provided in the Supplementary Information/Source Data file. The structures for DFT simulations are provided in the Supplementary Data file. These structures are visualized using Vesta (veresion 3.5.8) in this paper. Source data are provided with this paper.

Research involving human participants, their data, or biological material

Policy information about studies with human participants or human data. See also policy information about sex, gender (identity/presentation), and sexual orientation and race, ethnicity and racism.

Reporting on sex and gender	This study is not related to sex and gender since no human participants are involved in this study.
Reporting on race, ethnicity, or other socially relevant groupings	This study not is not related to race, ethnicity or other socially related groupings since no human participants are involved in this study.
Population characteristics	This study is not related to population characteristics since no human participants are involved in this study.
Recruitment	This is not related since no human participants are involved in this study.
Ethics oversight	This is not related since no human participants are involved in this study.

Note that full information on the approval of the study protocol must also be provided in the manuscript.

Field-specific reporting

Please select the one below that is the best fit for your research. If you are not sure, read the appropriate sections before making your selection.

☐ Life sciences ☐ Behavioural & social sciences ☒ Ecological, evolutionary & environmental sciences

For a reference copy of the document with all sections, see nature.com/documents/nr-reporting-summary-flat.pdf

Ecological, evolutionary & environmental sciences study design

All studies must disclose on these points even when the disclosure is negative.

Study description	This research focus on a reaction-agnostic framework based on high-throughput deep reinforcement learning with first principles (HDRL-FP) that offers excellent generalizability for investigating catalytic reactions. Using Haber-Bosch ammonia synthesis, we demonstrate the effectiveness of this framework.
Research sample	We focus on the hydrogen migration reaction of NH2 to NH3 on Fe surface since it is one of the most important reaction steps in Haber-Bosch reaction. We also examined the N/N2 diffusion on the Fe surface since they are related to important N2 adsorption/desorption steps. Our framework is demonstrated in both reactions. All the research are computational work.
Sampling strategy	We do the sampling when demonstrating the robustness and convergence of RL, as shown in Figure 2 and Figure 7, we select different numbers (e.g. n=4, 20, 100 and 500) of concurrent environment instances for the convergence of RL. These numbers were selected based on empirical testing. Our results (Figures 2 and 7) show that n=500 achieve good convergence within 60 mins computational time. For each environment instances (n), we did 5 independent runs to shows that the initial parameters of neural network do not affect the convergence. 5 runs were selected due to the reasonable standard deviation in both figure 2 and 6, especially for large n values.
Data collection	The data was collected from the computational simulations. The DFT simulations are not affected by any factors. The reinforcement learning simulations depend on the initial parameters of neural network. We did 5 independent simulations and demonstrate a good convergence (Figure 2 and 7) for large n values (> 20).
Timing and spatial scale	The data was collected when the simulations are done. The simulation results do not alter with time and spatial scale.
Data exclusions	No data was excluded from the analysis.
Reproducibility	All attempts to repeat the simulation results are successful. As mentioned above, the convergence time for RL depends on the initial parameters of neural network, but it converge well with large n values (>20).

Randomization	The initial parameters of the neural networks are randomized, which cause the statical errors in the RL simulations.
Blinding	No blinding was performed is this study. This is because the data are all acquired from computational modeling on chemical reactions.

Did the study involve field work? ☐ Yes ☒ No

Reporting for specific materials, systems and methods

We require information from authors about some types of materials, experimental systems and methods used in many studies. Here, indicate whether each material, system or method listed is relevant to your study. If you are not sure if a list item applies to your research, read the appropriate section before selecting a response.

Materials & experimental systems

n/a	Involved in the study
☒	☐ Antibodies
☒	☐ Eukaryotic cell lines
☒	☐ Palaeontology and archaeology
☒	☐ Animals and other organisms
☒	☐ Clinical data
☒	☐ Dual use research of concern
☒	☐ Plants

Methods

n/a	Involved in the study
☒	☐ ChIP-seq
☒	☐ Flow cytometry
☒	☐ MRI-based neuroimaging

Plants

Seed stocks	This is not related since no plants are involved in this study
Novel plant genotypes	This is not related since no plants are involved in this study
Authentication	This is not related since no plants are involved in this study
